# Supplementary material for: Parent-mediated interventions versus usual care in children with autism spectrum disorders: A protocol for a systematic review with meta-analysis and Trial Sequential Analysis
Source: PLoS One. 2025 May 16;20(5):e0323798. doi: 10.1371/journal.pone.0323798 (PMC12083817; doi:10.1371/journal.pone.0323798)
Supplement: S2 File — Search strategies for ‘Parent-mediated interventions versus usual care in children with autism spectrum disorders’. (PDF) [file pone.0323798.s002.pdf]

Search strategies for
'Parent-mediated interventions versus usual care in children with autism spectrum disorders'

Databases and results

Database	Platform	Results	Date
Medline	Ovid	4008	05.07.2024
Embase	Ovid	5289	06.07.2024
Cochrane Library	Wiley	4488	05.07.2024
Web of Science	Clarivate	622	05.07.2024
PsycINFO	APA	1453	05.07.2024
LILACS	Regional Portal of the Virtual Health Library (VHL)	1075	06.07.2024
All		16935	
÷ duplicates with EndNote		11121	

Ovid MEDLINE(R) and Epub Ahead of Print, In-Process, In-Data-Review & Other Non-Indexed Citations, Daily and Versions <1946 to July 03, 2024>

1	exp Child Development Disorders, Pervasive/	50965
2	exp Child Behavior/	27754
3	exp Language Development Disorders/	7569
4	(((autism or autistic or development* or neurodevelopment*) and disorder*) or ASD).mp.	419245
5	1 or 2 or 3 or 4	443570
6	exp Parent-Child Relations/	61719
7	exp Family Therapy/	9263
8	exp Behavior Therapy/	93108
9	exp Early Intervention, Educational/	3608
10	exp Adaptation, Psychological/	142417
11	exp Parents/	149554
12	exp Interpersonal Relations/	356181
13	exp Communication/px, th [Psychology, Therapy]	2481
14	(((parent* or caregiver*) adj3 (intervention* or interaction* or relation* or therap* or educat* or train* or mediated)) or PMI).mp.	96595
15	6 or 7 or 8 or 9 or 10 or 11 or 12 or 13 or 14	774240
16	5 and 15	46692
17	(baby or babies or infan* or child* or toddler* or pre-school* or preschool* or adolescen* or teen* or schoolchild* or school-child* or schoolage or school-age or paediatric* or pediatric*).mp.	4771156
18	16 and 17	38849
19	(randomized controlled trial or controlled clinical trial or retracted publication or retraction of publication).pt.	752414
20	clinical trials as topic.sh.	202734
21	(random* or placebo*).ab. or trial.ti.	1667754
22	19 or 20 or 21	2030043
23	exp animals/ not humans.sh.	5237020
24	22 not 23	1867451

25	18 and 24	4008

Embase <1974 to 2024 July 05>

1	exp *autism/	62800
2	exp *child behavior/	23012
3	exp *developmental language disorder/	1562
4	(((autism or autistic or development* or neurodevelopment*) and disorder*) or ASD).mp.	634495
5	1 or 2 or 3 or 4	668776
6	exp *child parent relation/	44988
7	exp *family therapy/	7159
8	exp *behavior therapy/	30311
9	exp *early childhood intervention/	1672
10	exp *psychological adjustment/	3971
11	exp *parent/	71700
12	exp *human relation/	310784
13	exp *interpersonal communication/	226378
14	(((parent* or caregiver*) adj3 (intervention* or interaction* or relation* or therap* or educat* or train* or mediated)) or PMI).mp.	139665
15	6 or 7 or 8 or 9 or 10 or 11 or 12 or 13 or 14	705261
16	5 and 15	38352
17	(baby or babies or infan* or child* or toddler* or pre-school* or preschool* or adolescen* or teen* or schoolchild* or school-child* or schoolage or school-age or paediatric* or pediatric*).mp.	4808230
18	16 and 17	32229
19	*Randomized controlled trial/ or *Controlled clinical study/ or *randomization/ or *intermethod comparison/ or *double blind procedure/ or *human experiment/ or *retracted article/	44433
20	(random$ or placebo or parallel group$1 or crossover or cross over or assigned or allocated or volunteer or volunteers).ti,ab.	2716303
21	(compare or compared or comparison or trial).ti.	1040028
22	((evaluated or evaluate or evaluating or assessed or assess) and (compare or compared or comparing or comparison)).ab.	2952327
23	(open adj label).ti,ab.	116471
24	((double or single or doubly or singly) adj (blind or blinded or blindly)).ti,ab.	284141
25	((assign$ or match or matched or allocation) adj5 (alternate or group$1 or intervention$1 or patient$1 or subject$1 or participant$1)).ti,ab.	436695
26	(controlled adj7 (study or design or trial)).ti,ab.	476076
27	(erratum or tombstone).pt. or yes.ne.	319993
28	or/19-27	6136892
29	(random$ adj sampl$ adj7 ('cross section$' or questionnaire$ or survey$ or database$1)).ti,ab. not (comparative study/ or controlled study/ or randomi?ed controlled.ti,ab. or randomly assigned.ti,ab.)	10045
30	*Cross-sectional study/ not (*randomized controlled trial/ or *controlled clinical study/ or *controlled study/ or randomi?ed controlled.ti,ab. or control group$1.ti,ab.)	14352

31	(((case adj control$) and random$) not randomi?ed controlled).ti,ab.	22549
32	(Systematic review not (trial or study)).ti.	290441
33	(nonrandom$ not random$).ti,ab.	19590
34	'Random field$'.ti,ab.	3087
35	(random cluster adj3 sampl$).ti,ab.	1660
36	(review.ab. and review.pt.) not trial.ti.	1209375
37	'we searched'.ab. and (review.ti. or review.pt.)	53571
38	'update review'.ab.	143
39	(databases adj4 searched).ab.	69311
40	(rat or rats or mouse or mice or swine or porcine or murine or sheep or lambs or pigs or piglets or rabbit or rabbits or cat or cats or dog or dogs or cattle or bovine or monkey or monkeys or trout or marmoset$1).ti. and animal experiment/	1258378
41	Animal experiment/ not (human experiment/ or human/)	2646788
42	or/29-41	4196966
43	28 not 42	5451939
44	18 and 43	5289

Cochrane Central Register of Controlled Trials Issue 7 of 12, July 2024

#1	MeSH descriptor: [Child Development Disorders, Pervasive] explode all trees	2900
#2	MeSH descriptor: [Child Behavior] explode all trees	2898
#3	MeSH descriptor: [Language Development Disorders] explode all trees	288
#4	(((autism or autistic or development* or neurodevelopment*) and disorder*) or ASD):ti,ab,kw	22678
#5	{OR #1-#4}	25247
#6	MeSH descriptor: [Parent-Child Relations] explode all trees	2705
#7	MeSH descriptor: [Family Therapy] explode all trees	1184
#8	MeSH descriptor: [Behavior Therapy] explode all trees	25045
#9	MeSH descriptor: [Early Intervention, Educational] explode all trees	704
#10	MeSH descriptor: [Adaptation, Psychological] explode all trees	6918
#11	MeSH descriptor: [Parents] explode all trees	8803
#12	MeSH descriptor: [Interpersonal Relations] explode all trees	8846
#13	MeSH descriptor: [Communication] explode all trees	13330
#14	(((parent* or caregiver*) near/3 (intervention* or interaction* or relation* or therap* or educat* or train* or mediated)) or PMI):ti,ab,kw	17596
#15	{OR #6-#14}	68233
#16	#5 AND #15	5240
#17	(baby or babies or infan* or child* or toddler* or pre-school* or preschool* or adolescen* or teen* or schoolchild* or school-child* or schoolage or school-age or paediatric* or pediatric*):ti,ab,kw	367367
#18	#16 AND #17 in Trials	4488

Web of Science: Science Citation Index Expanded (1900-present) and Conference Proceedings Citation Index – Science (1990-present)

#1	(((autism or autistic or development* or neurodevelopment*) and disorder*) or ASD) (Topic)	294353

#2	(((parent* or caregiver*) near/3 (intervention* or interaction* or relation* or therap* or educat* or train* or mediated)) or PMI) (Topic)	56487
#3	(baby or babies or infan* or child* or toddler* or pre-school* or preschool* or adolescen* or teen* or schoolchild* or school-child* or schoolage or school-age or paediatric* or pediatric*) (Topic)	2493846
#4	#1 AND #2 AND #3	3057
#5	(random* or blind* or placebo* or meta-analys* or trial*) (Title) OR (random* or blind* or placebo* or meta-analys*) (Topic)	3087460
#6	#4 AND #5	622

APA PsycInfo, APA PsycArticles

8	5 AND 6 AND Publication Type: Peer Reviewed Journal	1453
7	5 AND 6	1680
6	Index Terms: {Randomized Controlled Trials} OR {Clinical Trials} OR Title: trial OR Abstract: random* OR Abstract: placebo*	289915
5	3 AND 4	30523
4	Title: baby OR Title: babies OR Title: infan* OR Title: child* OR Title: toddler* OR Title: "pre-school*" OR Title: preschool* OR Title: adolescen* OR Title: teen* OR Title: schoolchild* OR Title: "school-child*" OR Title: schoolage OR Title: "school-age" OR Title: paediatric* OR Title: pediatric* OR Abstract: baby OR Abstract: babies OR Abstract: infan* OR Abstract: child* OR Abstract: toddler* OR Abstract: "pre-school*" OR Abstract: preschool* OR Abstract: adolescen* OR Abstract: teen* OR Abstract: schoolchild* OR Abstract: "school-child*" OR Abstract: schoolage OR Abstract: "school-age" OR Abstract: paediatric* OR Abstract: pediatric* OR Keywords: baby OR Keywords: babies OR Keywords: infan* OR Keywords: child* OR Keywords: toddler* OR Keywords: "pre-school*" OR Keywords: preschool* OR Keywords: adolescen* OR Keywords: teen* OR Keywords: schoolchild* OR Keywords: "school-child*" OR Keywords: schoolage OR Keywords: "school-age" OR Keywords: paediatric* OR Keywords: pediatric*	1129017
3	1 AND 2	36991
2	Index Terms: {Parent Child Relations} OR {Father Child Relations} OR {Mother Child Relations} OR {Parental Attitudes} OR Index Terms: {Family Therapy} OR {Conjoint Therapy} OR {Strategic Family Therapy} OR {Structural Family Therapy} OR Index Terms: {Early Intervention} OR {Coping Behavior} OR Index Terms: {Parents} OR {Adoptive Parents} OR {Expectant Parents} OR {Fathers} OR {Foster Parents} OR {Homosexual Parents} OR {Mothers} OR {Parental Characteristics} OR {Single Parents} OR {Stepparents} OR {Surrogate Parents (Humans)} OR Index Terms: {Interpersonal Relationships} OR {Caregivers} OR {Close Relationships} OR {Couples} OR {Family Relations} OR {Friendship} OR {Kinship} OR {Marital Relations} OR {Mentor} OR {Partners} OR {Peers} OR {Relationship Quality} OR {Relationship Termination} OR {Role Models} OR {Significant Others} OR Index Terms: {Interpersonal Communication} OR {Active Listening} OR {Apology} OR {Arguments} OR {Body Language} OR {Conversation} OR {Cross Cultural Communication} OR {Double Bind Interaction} OR {Eye Contact} OR {Gossip} OR {Group Discussion} OR {Intersubjectivity} OR {Interviewing} OR {Interviews} OR {Job Applicant Interviews} OR {Listening (Interpersonal)} OR {Negotiation} OR {Parent Child Communication} OR {Psychological Contracts} OR {Secrecy} OR {Self-Disclosure}	523016

	OR ((Title: parent* OR Title: caregiver*) NEAR/3 (Title: intervention* OR Title: interaction* OR Title: relation* OR Title: therap* OR Title: educat* OR Title: train* OR Title: mediated)) OR Title: PMI OR ((Abstract: parent* OR Abstract: caregiver*) NEAR/3 (Abstract: intervention* OR Abstract: interaction* OR Abstract: relation* OR Abstract: therap* OR Abstract: educat* OR Abstract: train* OR Abstract: mediated)) OR Abstract: PMI OR ((Keywords: parent* OR Keywords: caregiver*) NEAR/3 (Keywords: intervention* OR Keywords: interaction* OR Keywords: relation* OR Keywords: therap* OR Keywords: educat* OR Keywords: train* OR Keywords: mediated)) OR Keywords: PMI	
1	Index Terms: {Neurodevelopmental Disorders} OR {Attention Deficit Disorder} OR {Autism Spectrum Disorders} OR {Developmental Disabilities} OR {Disruptive Behavior Disorders} OR {Emotional and Behavioral Disorders} OR {Intellectual Development Disorder} OR {Learning Disorders} OR Index Terms: {Child Behavior} OR Index Terms: {Language Development} OR {Language Delay} OR {Reading Development} OR {Speech Development} OR {Delayed Speech} OR ((Title: autism OR Title: autistic OR Title: development* OR Title: neurodevelopment*) AND Title: disorder*) OR Title: ASD OR ((Abstract: autism OR Abstract: autistic OR Abstract: development* OR Abstract: neurodevelopment*) AND Abstract: disorder*) OR Abstract: ASD OR ((Keywords: autism OR Keywords: autistic OR Keywords: development* OR Keywords: neurodevelopment*) AND Keywords: disorder*) OR Keywords: ASD	276943

Latin American and Caribbean Health Sciences Literature (LILACS)

1	((((autism OR autistic OR development* OR neurodevelopment*) AND disorder*) OR asd)) AND ((((parent* OR caregiver*) AND (intervention* OR interaction* OR relation* OR therap* OR educat* OR train* OR mediated)) OR pmi)) AND ((baby OR babies OR infan* OR child* OR toddler* OR pre-school* OR preschool* OR adolescen* OR teen* OR schoolchild* OR school-child* OR schoolage OR school-age OR paediatric* OR pediatric*)) AND (db:("LILACS"))	1075
