# Supplementary material for: Parent-mediated interventions versus usual care in children with autism spectrum disorders: A protocol for a systematic review with meta-analysis and Trial Sequential Analysis
Source: PLoS One. 2025 May 16;20(5):e0323798. doi: 10.1371/journal.pone.0323798 (PMC12083817; doi:10.1371/journal.pone.0323798)
Supplement: Attachment — Submitted filename: Response to Reviewers.docx [file pone.0323798.s004.docx]

# Response to Reviewers

**Comments for the academic editor:**

1. I have edited the manuscript including file naming following the requirements from PLOS ONE.
2. I have added the following to the Coverletter:” This work was supported by The Lundbeck Foundation, grant number R389-2021-1597, TrygFonden, grant number 155050, Novo Nordisk Foundation, grant number NNF22SA0081180, Karen Elise Jensen Foundation, and the Simon Fougner Hartmann Family Fund. The funders had no role in study design, data collection and analysis, decision to publish, or preparation of the manuscript.”
3. It was misleading to state that I would make data available on acceptance, when I completed the data availability statement of the submission form. As this is a protocol for a future review there is no data. For the future review all data will be publicly available. The text should have been “No datasets were generated or analysed during the current study. All relevant data will be made available upon study completion”. I apologise for this inconvenience.
4. I have reviewed the Reference list and have found that it is complete and correct.

**Comments for reviewer #1**

Thank you so much for taking the time to review our protocol for a review with meta-analysis.

**Comments for reviewer #2**

Thank you so much for taking the time to review our protocol for a review with meta-analysis.

1. Thank you for pointing this out. “Usual care” is indeed a concept that has challenged us. It is widely used within the trials, as it seems unethical to limit children from receiving potential beneficial help, which they would have had, if they did not participate in the trial.

I have added the following to the Methods l. 274-276: “In usual care control groups parents are usually not restricted from having their child participate in other not parent-mediated interventions of their choice e.g., speech therapy or behavioural therapy.”

2. Thank you for suggesting further clarification of limitations for previous reviews. I decided to report on some of the items from the AMSTAR tool to demonstrate some of the limitations.

I have added the following to l. 207-213: “The reviews all had performed study selection in duplicate (AMSTAR item 5, Table 2) and used appropriate measures for statistical combination of results (item 11, Table 2). However, several of the other AMSTAR items were not fulfilled by some of the reviews, e.g., registration of a prespecified protocol (item 2, Table 2), providing a list of excluded studies and justifying exclusion (item 7, Table 2), reporting on sources for funding in included studies (item 10, Table 2), providing explanation for any heterogeneity observed in the results (item 14, Table 2).”

3. In our manuscript l. 342-350 we have the following statement regarding unpublished and grey literature: “We will consider accessing and reading conference abstracts from conferences in child and adolescent psychiatry and psychology, autism and autism intervention (e.g., ESCAP (European Society for Child and Adolescent Psychiatry) Congresses, Autism-Europe International Congress and INSAR (International Society for Autism Research). Additionally, we will consider relevant-for-the-review unpublished and grey literature trials, if identified. Finally, we will consult content experts for relevant studies.”
4. Thank you so much for pointing this out. We have now specified the age cut-offs for younger and older children in l. 544-545 “Younger (aged less than 7 years) versus older (with an age range including 7 years and older) children.”
